# Polymorphisms of *MTHFR* Associated with Higher Relapse/Death Ratio and Delayed Weekly MTX Administration in Pediatric Lymphoid Malignancies

**DOI:** 10.1155/2013/238528

**Published:** 2013-12-10

**Authors:** Hiroko Fukushima, Takashi Fukushima, Aiko Sakai, Ryoko Suzuki, Ryoko Nakajima-Yamaguchi, Chie Kobayashi, Atsushi Iwabuchi, Makoto Saito, Ai Yoshimi, Tomohei Nakao, Keisuke Kato, Masahiro Tsuchida, Hideto Takahashi, Kazutoshi Koike, Nobutaka Kiyokawa, Emiko Noguchi, Ryo Sumazaki

**Affiliations:** ^1^Department of Child Health, Faculty of Medicine, University of Tsukuba, 1-1-1 Tennodai, Tsukuba, Ibaraki 305-8575, Japan; ^2^Department of Pediatrics, University of Tsukuba Hospital, Japan; ^3^Department of Hematology/Oncology, Ibaraki Children's Hospital, Japan; ^4^Department of Epidemiology, Faculty of Medicine, University of Tsukuba, Japan; ^5^Department of Pediatric Hematology and Oncology Research, National Research Institute for Child Health and Development, Japan; ^6^Department of Genetics, Faculty of Medicine, University of Tsukuba, Japan

## Abstract

*Backgrounds*. Outcome of childhood malignancy has been improved mostly due to the advances in diagnostic techniques and treatment strategies. While methotrexate (MTX) related polymorphisms have been under investigation in childhood malignancies, many controversial results have been offered. *Objectives*. To evaluate associations of polymorphisms related MTX metabolisms and clinical course in childhood lymphoid malignancies. *Method*. Eighty-two acute lymphoblastic leukemia and 21 non-Hodgkin's lymphoma children were enrolled in this study. Four single nucleotide polymorphisms in 2 genes (*MTHFR* (rs1801133/c.677C>T/p.Ala222Val and rs1801131/c.1298A>C/p.Glu429Ala) and *SLCO1B1* (rs4149056/c.521T>C/p.V174A and rs11045879/c.1865+4846T>C)) were genotyped by Taqman PCR method or direct sequencing. Clinical courses were reviewed retrospectively. *Results*. No patient who had the AC/CC genotype of rs1801131 (*MTHFR*) had relapsed or died, in which distribution was statistically different among the AA genotype of rs1801131 (*P* = 0.004). Polymorphisms of *SLCO1B1* (rs11045879 and rs4149056) were not correlated with MTX concentrations, adverse events, or disease outcome. *Conclusions*. Polymorphisms of *MTHFR* (rs1801131) could be the plausive candidate for prognostic predictor in childhood lymphoid malignancies.

## 1. Introduction

Childhood cancer is a rare disease affecting 1 in 70,000 children aged 14 years and younger [1, 2]. Lymphoid malignancy, including leukemia and lymphoma, is the most common childhood cancer, accounting for 40% of all pediatric malignancies [2]. During the last 20 years, survival rates for pediatric acute lymphoblastic leukemia (ALL) and non-Hodgkin's lymphoma (NHL) have improved dramatically, mostly due to improvement of chemotherapy, allogeneic hematopoietic stem cell transplantation, and diagnostic techniques, with expected cure rates higher than 80% for pediatric lymphoid malignancy [1–3].

**Figure 1:**
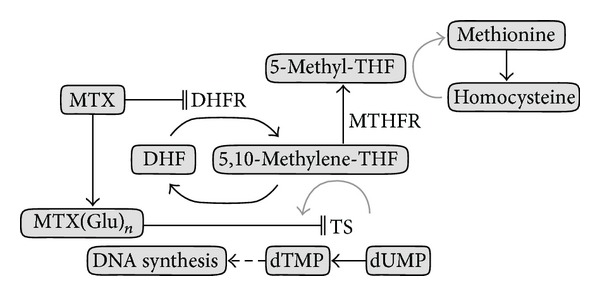
Schematic representation of methotrexate action. MTX inhibits folic metabolism through two mechanisms: MTX inhibits DHFR, which leads to the depletion of THF compounds, resulting in impairment of purine and thymidine synthesis. Polyglutamated MTX inhibits TS directly, which causes depletion of DNA synthesis. MTX: methotrexate; DHFR: dihydrofolate reductase; DHF: dihydrofolate; THF: tetrahydrofolate; TS: thymidylate synthase; MTHFR: methylenetetrahydrofolate reductase; MTX (Glu)_*n*_: MTX glutamates; dTMP: deoxythymidine monophosphate; dUMP: deoxyuridine monophosphate.

Methotrexate (MTX) is one of the key drugs for cancer treatment and a proven critical component for pediatric ALL and NHL [1, 4, 5]. MTX interrupts the folic acid cycle by inhibiting two enzymes (Figure 1). Firstly, as an analog of folate, MTX is a powerful competitive inhibitor of dihydrofolate reductase (DHFR) [6, 7]. DHFR is responsible for converting folates to their active form tetrahydrofolate, a substrate of thymidylate synthase (TS), to convert deoxyuridine monophosphate to deoxythymidine-5′-monophosphate resulting in DNA synthesis. Secondly, the polyglutamated forms of MTX inhibit the activity of TS directly [6, 8]. High-dose MTX (HD-MTX) treatment has been proven for its efficacy for the treatment of ALL and NHL [4, 5]. However, MTX often causes toxicity such as renal failure, hepatotoxicity, and severe mucositis requiring a dose reduction and cessation of treatment or hemodialysis, and it is well known that large interindividual MTX kinetic variability exhibits [9]. Therefore, it is beneficial to find patients with a high risk of developing adverse events before the initiation of the treatment [9, 10]. In folate metabolism, methylenetetrahydrofolate reductase (MTHFR) is a key molecule to convert 5,10-methylenetetrahydrofolate (5,10-methylene-THF) to 5-methyltetrahydrofolate (5-methyl-THF), and 5,10-methylene-THF is a substrate of TS [11]. There are two extensively examined *MTHFR* polymorphisms, rs1801133 (c.677C>T, p.Ala222Val) and rs1801131 (c.1298A>C, p.Glu429Ala), that have been shown to have lower enzyme activity when they carry mutant alleles [12].

A previous study reported that the T allele of *MTHFR* rs1801133 was associated with an increased risk of relapse events but not associated with MTX concentrations nor adverse events and that rs1801131 was not associated with altered risks of relapse nor toxicity in 520 pediatric ALL patients by the Children's Oncology Group [13]. Others showed conflicting results, showing that patients with the TT genotype of *MTHFR* rs1801133 resulted in a better overall survival rate in 126 Brazilian pediatric ALL patients treated with MTX [14], and others reported that patients carrying the T allele of rs1801133 and the A allele of rs1801131 (*MTHFR* c.T677A1298 haplotype) had a lower event free survival [15], and T allele of *MTHFR* rs1801133 and C allele of *MTHFR* rs1801131 had higher relapse ration [16].

Transporters such as adenosine triphosphate-binding cassette (ABC) transporters and organic anion transporters were also reported to act for MTX disposition [17]. Solute carrier organic anion transporter family member 1B1 (SLCO1B1) is one of the organic anion transporters and localized at the sinusoidal membrane of hepatocytes, and its transcript has been detected in enterocytes. *SLCO1B1* transfected cells were proven to uptake MTX in vitro, as well as other compounds such as estradiol, bilirubin, bile acids, 3-hydroxy-3-methylglutaryl coenzyme A (HMG-CoA), rifampicin, angiotensin-converting enzyme inhibitors, and the active metabolite of irinotecan, SN-38 [18–20]. Tirona et al. reported *SLCO1B1* polymorphisms (rs56101265, rs5606188, rs4149056, rs55901008) altered in transport of substrates in vitro [19]. Kameyama et al. reported *SLCO1B1* polymorphisms rs4149056 (c.521T>C, p.V174A) transfected HEK cells, decreasing transporting activities [21]. Children who underwent HD-MTX for ALL who carry *SLCO1B1* polymorphism rs11045879 (c.1865+4846T>C) and rs4149056 were shown to have lower MTX elimination in a genome-wide-association-study (GWAS), whose cohort mostly consisted of Caucasians and a limited number of Asians [20, 22]. These polymorphisms in *SLCO1B1* were confirmed associations with a higher MTX concentration at 72 hrs among Spanish B-ALL children [22, 23]. Japanese patients treated with MTX due to rheumatoid arthritis showed no association between *SLCO1B1* polymorphisms (rs4149056 and rs2306283 (c.388A>G, p.N432D)) and MTX concentration nor disease status [24].

Although many studies have been conducted to investigate associations between *MTHFR* polymorphisms and MTX toxicity in pediatric ALL patients, results derived from these studies are conflicting. Study populations were mostly Caucasians and reports from Asians were limited [10, 25]. Allele frequencies of polymorphisms differ in each ethnic population, and their effects can be influenced by different chemotherapy protocols, genetic backgrounds, and others [13, 26, 27].

In the present study, we genotyped 2 polymorphisms in *MTHFR* (rs1801133 and rs1801131) and 2 more polymorphisms in *SLCO1B1* (rs4149056 and rs11045879) in Japanese ALL/NHL patients treated with HD-MTX and examined the relationship between genotypes and prognosis/adverse events.

## 2. Method

### 2.1. Objectives

To investigate associations of polymorphisms of *MTHFR* (rs1801133 and rs1801131) and *SLCO1B1* (rs4149056 and rs11045879) and prognosis or clinical course including MTX concentrations in Japanese children who developed lymphoid malignancy treated with HD-MTX.

### 2.2. Patients

Eighty-two acute lymphoblastic leukemia (ALL) and 21 non-Hodgkin lymphoma (NHL) children were enrolled in the present study. All patients were treated at two main and exclusively pediatric malignancy treating regional hospitals in Ibaraki prefecture (University of Tsukuba Hospital and Ibaraki Children's Hospital) between November 1993 and November 2012. Patients' characteristics are shown in Table 1.

Informed consent was obtained from each parent/guardian or patient. The study was approved by the ethics committee of the University of Tsukuba in accordance with the Ethical Guidelines for Human Genome/Gene Analysis Research of the Ministry of Health, Labor and Welfare of Japan and the Declaration of Helsinki.

### 2.3. Treatment, Methotrexate Concentration, and Toxicity Evaluation

All patients received intravenous MTX continuously at 3 g/m^2^/12 hrs or 24 hrs, or 5 g/m^2^/24 hrs with folic acid rescue following the protocol of the Tokyo Children's Cancer Study Group (TCCSG) L99-15 [28], ALL-BFM 95 [29], or NHL B9604 [30].

Monitoring of MTX concentration in plasma was carried out every day until the concentration was below 0.1 *μ*mol/L.

Toxicity data were retrospectively collected objectively, blinded genotypes, from the patients' medical files. Toxicity was graded according to the Common Terminology Criteria for Adverse Event (CTCAE) v4.0 released from the Cancer Therapy Evaluation Program of the National Cancer Institute (http://ctep.cancer.gov/). The highest grade of toxicity observed for each patient during the MTX therapy period was recorded. Data were collected including vomiting, diarrhea, serum hepatic enzyme (ALT), serum bilirubin and renal toxicity (serum creatinine), and MTX concentrations at 48 and 72 hrs after infusion. MTX levels were considered high if the concentration was above 1.0 *μ*mol/L at 48 hrs or 0.1 *μ*mol/L at 72 hrs. Above 1.5 mg/dL was considered as hyperbilirubinemia.

Patients who developed at least one adverse event (maximum/base line creatinine ratio higher than 1.5, ALT elevation more than grade 1 according to the criteria of CTCAE v.4.0, T-Bil elevation ≥1.5 mg/dL) were categorized as having global toxicity.

### 2.4. Genotyping

DNA was extracted using Genomic DNA Isolation Kit (QiAamp DNA Blood Mini Kit, QiAamp DNA Blood Midi Kit or QIAamp DNA FFPE Tissue: Qiagen, Vealo, The Netherlands) from 0.5 to 2 mL peripheral blood, bone marrow, or paraffin-embedded bone marrow/tissue in complete remission following the manufacturer's instructions.

Polymorphisms of *MTHFR* (c.677C>T, rs1801133, c.1298A>C, rs1801131) and *SLCO1B1* (c.1865+4846T>C, rs11045879, c.521T>C, rs4149056) were genotyped using the TaqMan Assay-on-Demand SNP Typing System (Applied Bio Systems, Foster City, CA, USA) following the manufacturer's instructions. PCR was performed on a 384-well format with 5 ng of DNA each, and automatic allele calling was performed using ABI PRISM 7900HT data collection and analysis software, version 2.2.2 (Applied Biosystems).

The accuracy of the genotyping for rs1801131 in children who had relapsed or died was confirmed by direct sequence using the primers 5′-TTTGGGGAGCTGAAGGACTA-3′ (forward) and 5′-CTTTGTGACCATTCCGGTTT-3′ (reverse) as reported by Shimasaki et al. [31] and the BigDye Terminator v.1.1 Cycle Sequencing Kit (Applied Biosystems) on an ABI PRISM 3130 Genetic Analyzer (Applied Biosystems).

### 2.5. Statistics

Deviation from Hardy-Weinberg expectations was examined by chi-square test. Control genotype frequencies were obtained from HapMap database (HapMap JPT, http://www.ncbi.nlm.nih.gov/) and Genome Medicine Database of Japan (GeMDBJ, https://gemdbj.nibio.go.jp/dgdb/index.do) which consisted of healthy Japanese individuals and have been genotyped using Illumina Sentrix HumanHap550 Genotyping BeadChip and InfiniumHD 610-Quad BeadChip (Illumina, San Diego, CA), and principal component analysis has been performed to remove relatives, duplications, and Han Chinese (https://gemdbj.nibio.go.jp/dgdb/web/common/Help.jsp#manual). The genetic effects of the association between the case-control status and each individual SNP were assessed by chi-square test or Fisher's exact test.

For the analysis of associations among adverse events during MTX treatment and polymorphisms, the worst value of toxicity markers (ALT elevation, bilirubin elevation, or creatinine ratio) that each patient experienced during all HD-MTX courses and polymorphisms of *MTHFR* and *SLCO1B1* were assessed by chi-square test.

To confirm the suitability of MTX concentrations as a toxicity marker, the associations between different toxicity markers (ALT elevation, bilirubin elevation, or creatinine ratio) and MTX plasma concentration at 48 and 72 hrs among evaluable 149 courses conducted for ALL in total were examined by chi-square test or Fisher's exact test.

Analysis was conducted using SPSS, version 12.0 (SPSS Inc. Chicago, IL, USA). A *P* value <0.05 was considered statistically significant.

## 3. Results

Children enrolled in our study consisted of 82 patients diagnosed as ALL (B-cell precursor 69, T-lineage 9, others 4) and 21 children diagnosed as NHL (Burkitt 10, T-lineage 5, Diffuse large B cell lymphoma 2, others 4). The number of patients who were in their first complete remission was 89, relapsed 8, and died 6. The average age at diagnosis was 7.4 (0.2–19.2) years old. There were 62 boys and 41 girls. Patients' characteristics are shown in Table 1.

Table 2 shows the genotype of *MTHFR* and *SLCO1B1* in patients and controls Hapmap JPT obtained from the NCBI database and GeMDBJ. Deviation from Hardy-Weinberg expectations was examined by chi-square test and was applied to each of SNPs (*P* = 0.049 for rs1801133, *P* = 0.633 for rs1801131, *P* = 0.495 for rs11045879, and *P* = 0.686 for rs4149056). Differences of allele frequencies were assessed by chi-square test and there were no differences with control data. Among Hapmap JPT, the *P* value for rs1801133 was 0.442, rs1801131 was 0.924, rs11045879 was 0.896, and rs4149056 was 0.066. Among GeMDBJ, the *P* value for rs1801133 was 0.885, rs1801131 was 0.765, and rs4149056 was 0.408.

**Figure 2:**
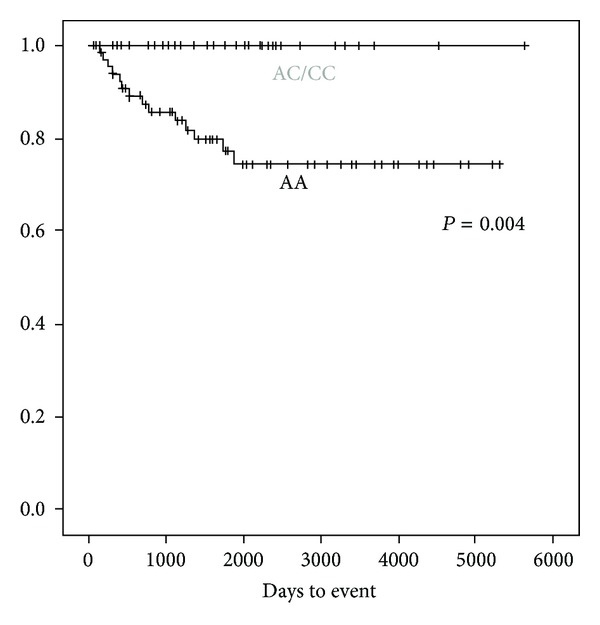
Event free survival according to *MTHFR* c.1298AA or AC/CC. Figure shows event free survival according to *MTHFR* c.1298A>C.

The outcome and each of polymorphisms are shown in Table 3. Thirty-four of 89 children in their first complete remission carried at least one C allele of rs1801131 (*MTHFR* c.1298AC/CC genotype), and no children who relapsed/died carried this genotype, in which frequency was significantly different (*P* = 0.004).  Figure 2 shows event free survival according to the genotype of *MTHFR* c.1298A>C (rs1801131).

All assessable weekly 3 g/m^2^ HD-MTX courses following the treatment protocol of TCCSG L99-15 for standard or high risk ALL patients totaled 149. For assessing the associations of adverse events during each MTX administration and each genotype, we analyzed these homogeneously treated 149 courses of weekly HD-MTX (Table 4). Courses undergone for children with the AA genotype of rs1801131 (*MTHFR* c.1298AA) tended to correlate to MTX concentration of higher than 1.0 *μ*mol/L at 48 hrs after administration but not statistically significant (*P* = 0.06), and no other genotypes were correlated to MTX concentrations.

Only 9 of 49 courses undertaken for children with the CC genotype of rs1801133 (*MTHFR* c.677CC) developed Common Terminology Criteria for Adverse Event (CTCAE) v4.0 more than grade 1 ALT elevation, whereas 41 of 100 courses undertaken for children with at least one T allele of rs1801133 (*MTHFR* c.677CT/TT) developed elevated serum liver enzyme (*P* = 0.006). Thirty-eight of 96 courses undertaken for children with the AA genotype of rs1801131 (*MTHFR* c.1298AA) resulted in elevated serum liver enzyme, 11 of 52 with at least one C allele of rs1801131 (*MTHFR* c.1298AC/CC genotype) resulted in ALT elevation (*P* = 0.036). Only 8 of 32 courses conducted with patients who carried the CC genotype of rs1801133 (*MTHFR* c.677CC) resulted in the next MTX administration being delayed more than 5 days due to adverse events such as serum hepatic enzyme, renal toxicity, or infection, as 33 of 69 courses for patients who carry at least one T allele of rs1801133 (*MTHFR* c.677CT/TT genotype) resulted in the next MTX administration being delayed which had been planned to have been conducted weekly (*P* = 0.030).

No adverse events such as elevated MTX concentration, ALT elevation, hyperbilirubinemia, or prognosis were statistically correlated to two polymorphisms in *SLCO1B1* (rs11045879 and rs4149056) (Tables 2 and 3).

MTX concentrations and adverse events were analyzed by chi-square test (Table 5). Elevated MTX concentrations at 48 hour were associated with higher creatinine ratio (*P* < 0.001) and delayed weekly MTX administration (*P* = 0.013).

## 4. Discussion

High dose methotrexate is highly effective for broad cancers including lymphoid malignancies such as leukemia and lymphoma. However, its adverse events differ among each ethnicity and could significantly affect its clinical course. Previously, genetic polymorphisms of MTX pathway protein were reported as one of the predictable values.

With recent excellent advances in the prognosis among childhood lymphoid malignancy, the rate of patients who relapsed or died is very limited. It is vitally important to detect the factors that influence the prognosis in this limited number of poorer prognostic patient cohort other than factors that have already been thoroughly examined. In our study, all children who had relapsed or died carried the AA genotype of rs1801131 (*P* = 0.004). Previous studies using Caucasian ALL patients reported that the T allele of rs1801133 was correlated with poor prognosis, but the polymorphism of rs1801131 was not associated with the prognosis of the patients [13]. On the contrary, the TT genotype of rs1801133 and the AC genotype of rs1801131 were reported to be associated with lower overall survival in Brazilian patients with pediatric ALL [14], and the T allele of rs1801133 and the A allele of rs1801131 (*MTHFR* c.T677A1298 haplotype) had a lower event free survival among Caucasians [15].

Substitution of A to C of rs1801131 causes an amino acid change of glutamine to alanine at the position 429 in* MTHFR* and showed lower enzyme activity in vitro [12], and lymphoblasts with the AC genotype of rs1801131 required higher MTX concentrations in order to inhibit 50% of the control TS activity than those with the CC genotype of rs1801131 [32]. Our results showing the AA genotype of rs1801131 to be associated with poor prognosis might be explained by the fact that lower MTHFR enzyme activity due to substitution of A to C of rs1801131 leads to reduced conversion of 5,10-methylene-THF to 5-methyl-THF, resulting in more substrate for TS, thereby leading to more DNA synthesis and lesser MTX sensitivity as reported in a previous ex vivo study [32].

Previously, rs1801133 (*MTHFR* c.677C>T) was reported as a candidate predictor of MTX lower elimination, but its results are controversial and many different results have been reported [10, 11, 23]. Trevino et al. discovered significant associations among lower MTX elimination and polymorphisms in *SLCO1B1* (rs11045879 and rs4149056). Lopez-Lopez et al. confirmed this result in Spanish population [20, 23]. Most of those researches were conducted among Caucasians and studies among Japanese were limited [24]. In our study population, all patients consisted of Japanese, and association study among *SLCO1B1* polymorphism in pediatric lymphoid malignancies had not been conducted previously.

MTX concentration at 48 hrs was one of the strong predictors to develop a delay in the next course of MTX administration and serum creatinine elevation (Table 5). In our study population, courses conducted for patients carrying the AA genotype of rs1801131 (*MTHFR* c.1298AA) tended to have a higher MTX concentration at 48 hrs; however, this association did not reach statistical significance. Polymorphisms in *SLCO1B1* (rs11045879 and rs4149056) were not correlated to MTX concentrations at 48 hrs (*P* = 0.687 and *P* = 0.567, resp.). The discrepancies among studies previously conducted among Caucasians might be caused by differences of genetic backgrounds, lifestyles, or treatment protocol. This is retrospective study, so in the future a prospective research for Japanese children is warranted.

Polymorphisms in *MTHFR* (rs1801133 and rs1801131) were associated with serum ALT elevation (*P* = 0.006 and *P* = 0.036, resp.). Patients who carried at least one T allele of rs1801133 (*MTHFR* c.677CT/TT genotype) were also associated with more than 5 days of delay of the next MTX administration, which was planned to undergo weekly (*P* = 0.03). Patients with adult acute lymphoblastic leukemia carrying at least one T allele of rs1801133 (*MTHFR* c.677CT/TT genotype) were associated with developing ALT elevation as CTCAE v.4.0 grade more than 1, which was the same result as reported previously [33]. However, this result is different from the two previous studies that were conducted for Japanese children with leukemia or lymphoma and did not show statistical associations between the T allele of rs1801133 and hepatic enzyme elevations [10, 31]. Chiusolo et al. reported that the AC genotype of rs1801131 (*MTHFR* c.1298 AC) showed lower adverse event [8]. de Jonge et al. reported that lymphoblast with the AC genotype of rs1801131 (*MTHFR* c.1298AC) required higher MTX concentrations in order to inhibit 50% of the control TS activity ex vivo [32]. These results suggest that the AA genotype of rs1801131 tends to develop more adverse events, which corresponds to our results.

Significant correlations of polymorphisms in *SLCO1B1* and MTX eliminations were discovered by the GWAS study, which mostly consists of ancestry of European or African descent and a small number of Asian descent [20, 23]. These polymorphisms were not correlated to MTX concentrations at 48 hrs in our study population (*P* = 0.529 for rs11045879, *P* = 0.413 for rs4149056) (Table 4). The differences might be caused from different protocols, ethnicity, or backgrounds, including supportive care or lifestyle and study design.

## 5. Conclusion

We conducted an association study of polymorphisms (rs1801133 and rs1801131 of *MTHFR* and rs11045879 and rs4149056 of *SLCO1B1*) and adverse events during HD-MTX treatment and prognosis in Japanese childhood lymphoid malignancies.

Patients that carried polymorphisms of rs1801131 (*MTHFR* c.1298AC/CC genotype) had not relapsed nor died. The CT/TT genotype of rs1801133 (*MTHFR* c.677CT/TT genotype) resulted in higher serum hepatic enzyme and delayed administrations during weekly high dose MTX.

Relations of *SLCO1B1* polymorphisms (rs11045879 and rs41490586) in Japanese children were assessed for the first time and found to have no correlations with any adverse events, MTX concentrations or outcome.

## Figures and Tables

**Table 1:** Patient characteristics.

Diagnosis	ALL	BCP	69
		T	9
		Other	4
	NHL	Burkitt	10
		DLBCL	2
		T	5
		Other	4

1CR/relapsed/dead			89/8/6

Sex	M : F		62 : 41

Age at diagnosis (y)			7.43 (0.2–19.2)

ALL: acute lymphoblastic leukemia; NHL: non-Hodgkin's lymphoma; BCP: B-cell precursor leukemia; T: T-cell linage; CR: complete remission.

**Table 2:** Allele frequencies.

Gene	Reference SNP ID	Genotype	Our study (*n* = 103)	HWE *P*	HapmapJPT^†^ (*n* = 172)	*P*	GeMDBJ^‡^(*MTHFR* c.677C>T: *n* = 2,375 *MTHFR* c.1298A>C: *n* = 1,428 *SLCO1B1 *c.521T>C: *n* = 1,427)	*P*
*MTHFR* c.677 C>T	rs1801133	CC	32		68		864	
		CT	61		84		1123	
		TT	10	0.049	20	0.442	388	0.885

*MTHFR* c.1298 A>C	rs1801131	AA	68		112		952	
		AC	31		56		435	
		CC	4	0.633	4	0.924	41	0.765

*SLCO1B1 *	rs11045879	TT	42		70		n.d.	
		TC	43		74		n.d.	
		CC	18	0.495	28	0.896	n.d.	n.d.

*SLCO1B1* c.521 T>C	rs4149056	TT	73		138		1048	
		TC	26		30		347	
		CC	4	0.686	4	0.066	32	0.408

Difference of each allele frequency was calculated by  *χ* ^2^  test.

^†^NCBI Hapmap JPT: database at National Center of Biotechnology Information (http://www.ncbi.nlm.nih.gov/).

The submitted SNP numbers to NCBI were ss65837366 for* MTHFR* C677T, ss76885974 for *MTHFR* A1298C, ss15510724 for *SLCO1B1* rs11045879, and ss105439952 for *SLCO1B1* T521C.

^‡^GeMDBJ: Genome Medicine Database of Japan (https://gemdbj.nibio.go.jp/dgdb/index.do).

**Table 3:** Outcome according to each genotype in all patients.

Gene	Locus or reference SNP ID		Patients number according to genotype	Relapsed/died of disease
*MTHFR *	C677T	CC	32	5
		CT/TT	70	8
			*P*	0.540
*MTHFR *	A1298C	AA	67	13
		AC/CC	35	0
			*P*	0.004
*SLCO1B1 *	rs11045879	TT	42	6
		TC/CC	60	7
			*P*	0.696
*SLCO1B1 *	T521C	TT	73	8
		TC/CC	29	5
			*P*	0.511
*MTHFR *	677CT/TT and 1298AA	no	18	0
		yes	53	8
			*P*	0.105

All patients who had relapsed or died of disease had *MTHFR* c.1298AA genotype.

**Table 4:** Clinical adverse event, delayed course, and polymorphisms during MTX 3 g/m^2^ courses in total 149.

Gene and locus or reference SNP ID	Number of courses according to genotype		MTX concentration (*µ*mol/L)		Creatine elevation	ALT elevation	T Bil (mg/dL) ≥1.50	Assessable course for duration	Delayed course (more than 5 days)
			at 48 hours ≥1.00	at 72 hours ≥0.10					
*MTHFR* C677T	CC	49	4	17	8	9	11	32	8
	CT/TT	100	11	32	11	41	15	69	33
		*P*	0.774	0.742	0.360	0.006	0.272	*P*	0.030
*MTHFR* A1298C	AA	96	13	36	14	38	13	67	30
	AC/CC	53	2	13	5	12	13	34	11
		*P*	0.060	0.107	0.367	0.036	0.096	*P*	0.230
*SLCO1B1* rs11045879	TT	66	6	21	9	25	11	45	18
	TC/CC	83	9	28	10	25	15	56	23
		*P*	0.687	0.805	0.773	0.319	0.796	*P*	0.913
*SLCO1B1* T521C	TT	100	9	30	12	35	14	68	28
	TC/CC	49	6	19	7	15	12	33	13
		*P*	0.567	0.284	0.694	0.594	0.120	*P*	0.864
*MTHFR* 677CT/TT and 1298AA	no	27	1	9	3	5	27	18	5
	yes	74	10	28	9	34	73	53	27
		*P*	0.279	0.678	1.000	0.012	0.194	*P*	0.088

All assessable HD-MTX (3 g/m^2^) courses undergone for leukemia were 149 in total. *MTHFR* c.677CT/TT and c.1298AA genotype were associated to hepatotoxicity. ALT assessed as elevated as CTCAE more than grade 1. Elevated creatinine was evaluated with increased serum creatinine more than 1.5 times compared with the value just before the MTX administration.

**Table 5:** MTX concentrations and ALT/Cre/T-Bil elevations evaluated for 147 courses in total.

MTX serum concentrations		Number of courses according to MTX concentrations	Creatinine ratio >1.5	ALT elevation CTCAE Grade more than 1	Bilirubin elevation >1.5 (mg/dL)	Assessable course for duration	Delayed duration (more than 5 days)
48 hour	<1.0 *μ*mol/L	132	10	46	20	87	31
	>1.0 *μ*mol/L	15	8	3	5	12	9
		*P*	0.000	0.248	0.138	*P*	0.013
72 hours	<0.1 *μ*mol/L	100	7	34	13	61	22
	>0.1 *μ*mol/L	49	12	16	13	41	19
		*P*	0.003	0.870	0.044	*P*	0.252

One MTX concentration less than 0.1 μmol/L at 48 hours.

Higher MTX concentration at 48 hours were associated to creatinine ratio and bilirubin elevation and MTX concentration at 72 hours were associated with creatinine elevation. No associations between MTX concentration and ALT elevations were found. One patient developed renal toxicity needing hemodialysis whose MTX concentration at 48 hours was 42.71 *μ*mol/L, worst creatinine was 11.9 times higher than before treatment, worst serum ALT was 77 IU/L and total bilirubin was 1.7 mg/dL. This patient never underwent another HD-MTX treatment again.
